# Indoor air microbial load, antibiotic susceptibility profiles of bacteria, and associated factors in different wards of Arba Minch General Hospital, southern Ethiopia

**DOI:** 10.1371/journal.pone.0271022

**Published:** 2022-07-07

**Authors:** Gebre Kayta, Aseer Manilal, Dagimawie Tadesse, Munira Siraj

**Affiliations:** Department of Medical Laboratory Science, College of Medicine and Health Sciences, Arba Minch University, Arba Minch, Ethiopia; Pondicherry University, INDIA

## Abstract

The levels of indoor air microbial load in hospitals are very crucial to the health of patients and health care workers and are to be regularly monitored and maintained at an acceptable level. However, this problem remains overlooked, particularly in developing countries including Ethiopia. A hospital-based cross-sectional study is designed to determine the indoor air microbial load (settle plate technique), microbial isolates (standard microbiological techniques), bacterial susceptibility profiles (Kirby-Bauer disk diffusion technique), and associated factors, in different wards of the title Hospital, southern Ethiopia. An observational checklist was used to collect relevant information related to the associated factors; descriptive and inferential statistics were applied using Statistical Package for Social Sciences (SPSS); p-values ≤ 0.05 in the multivariable analysis were considered statistically significant. The total average bacterial and fungal load of the selected wards was 1914±1081.4 Colony Forming Units (CFU)/m^3^ (95% CI: 1718.5–2109.48 CFU/m^3^) and 1533.7±858.8 CFU/m^3^ (95% CI: 1378.5-1688CFU/m^3^) respectively. The highest mean bacterial (1914±1081.4 CFU/m^3^) and fungal (1533.7±858.8 CFU/m^3^) loads were found in the male surgical and female medical wards respectively. A total of 229 bacterial and 139 fungal isolates were obtained; Gram-positive bacteria were the predominant type, 130 (56.7%), particularly the isolates of *Staphylococcus aureus*, 46 (20.1%). The predominant fungal isolates were *Aspergillus* sp., 53(38%). Percentages of multidrug-resistant (MDR), extended-spectrum beta-lactamase (ESBL), and carbapenemase producers respectively were 48.5, 26.5, and 25%. High room crowd index [p = 0.003; Adjusted Odds Ratio (AOR) 12.5 (Confidence Interval (CI) 95%: 2.42–65)], presence of damp/wet materials [p = 0.025; AOR 7 (CI 95%: 1.3–37.4)], intense room traffic [p = 0.004; AOR 9.6 (CI 95%: 1.2–79.3)], inappropriate storage of food and drugs [p = 0.008; AOR 7.5 (CI 95%: 1.7–32)], and unclean environment [p = 0.03; AOR 5.8 (CI 95%: 1.2–28)] showed statistical significance concerning the indoor air microbial loads; most of the wards in Arba Minch General Hospital (AMGH) stand high and not in an acceptable level as per the WHO and the European Commission standards on indoor air microbial load. Periodic air surveillance and infection prevention control programs are required to reduce the transmission of these microbes to inpatients, visitors, and health care workers.

## Introduction

A vast majority of microbes in the indoor air of healthcare facilities are generally considered innocuous, however, a fraction of them can be pathogenic and can cause several types of infections [1]. Bacteria and fungi are notorious in this context and they produce toxins (endotoxin and mycotoxin) and also they cause severe problems in the hospital environment, particularly in operating rooms, intensive care units, and neonatal wards [2]. This is critical in many instances and causes health problems in hospital occupants, particularly among vulnerable groups (older and immune-comprised ones) [3, 4]. In addition, the evolution and emergence of bacterial resistance to antibiotics and disinfectants act as added factors. Resistant microorganisms which remain air-suspended in the hospital environment are much more likely to be inhaled by different individuals, including patients [5]. The most threatening of these microbes are those that have the potential to spread by air-matrix and include Methicillin-Resistant *S*. *aureus* (MRSA), gentamicin-resistant Gram-negative bacteria, and multidrug-resistant Mycobacterium tuberculosis [6]. Therefore, determining the indoor air microbial load and antibiotic resistance of bacteria in healthcare settings are important from an epidemiologic perspective as well as in the context of maintaining proper health and safety of patients and healthcare workers.

The exact burden of diseases associated with contaminated indoor air in hospitals remains uncertain because of the difficulty to accumulate reliable data. At the same time, indoor air pollution caused by dampness, mold, chemicals, and other biological agents is the major factor contributing to morbidity and mortality worldwide [7]. Above all, it is reported that the most dreadful pathogens causing nosocomial infections are those that have the potential to spread by the air matrix [8]. Healthcare settings particularly in under-developed countries are breeding grounds for microorganisms due to overcrowding, improper building design, and poor ventilation [9]. Nowadays, the evaluation of the level of microbial contamination in air in hospitals is considered to be a basic and important step towards the prevention of airborne nosocomial infections [10]. However, less developed countries including Ethiopia have limited facilities and modalities to achieve this target, and there exist only less frequent assessments and monitoring schemes [11]. Studies so far performed in this line in Ethiopia have merely focused on determining the overall bacterial load and its types [9, 12]. The results obtained so far prove that the indoor air microbial load is above the sanitary standards set by European Commission for non-industrial premises and is not acceptable at all [13]. There are also shreds of evidence that suggest that the microbial load in the indoor air in hospital rooms varies among hospitals as well as from ward to ward in a particular hospital [12, 14]. Therefore, this study was conducted to assess the microbial load, its associated factors, and antibiotic-resistant patterns of bacteria in the indoor air of different patient admission wards of AMGH, Arba Minch, southern Ethiopia.

## Materials and methods

### Study area

The study was conducted at AMGH situated in Arba Minch town from 1^st^ February to 30^th^ April 2021; this town is located 505 km away from Addis Ababa in the southern part of Ethiopia. It has three government health institutions; one General Hospital and two Health Centers (Sikela and Secha). Arba Minch General Hospital, our study site is the biggest among these three and provides services to the residents of Gamo Zone, having 300 beds with a total of 757 workers. The average number of patients attending the health service amount to 120,000 per year, 10,000 per month, and 300 per day (2020 data, Health Management, Information System of AMGH) [15]. This hospital has departments including inpatient wards (surgical, pediatric, neonatal intensive care unit, intensive care unit (ICU), medical, gynecology and obstetrics), outpatient, emergency rooms, ophthalmology, anti-retroviral treatment room, tuberculosis ward, operation room, and laboratory facilities.

### Study design

We purposely selected inpatient wards, such as operation room (OR), surgical (SW), ICU, medical (MW), pediatrics (PW), orthopedic (OPW), and gynecology wards (GW). The inclusion and exclusion criteria respectively are rooms that are occupied by one or more patients, in the above wards and unoccupied inpatient rooms, along with office rooms.

### Sample size determination and sampling technique

A total of 240 settle plate samples, in two different media (ie., 120 SBA plates for bacteria and 120 SDA plates for fungi) were collected separately from ten rooms, belonging to seven inpatient wards, within three months consecutively, ie., twice per month and twice a day, making it a total of twenty samples per day (from ten rooms of selected wards, ie., 10×2 = 20). Since the samples were collected six times within three months (ie., 12 weeks), the total sample size reached two hundred and forty (2x20×6 = 240), comprising both bacteria and fungi. The sample size was calculated according to the number of rooms in AMGH, the allotted study period, and the facilities available.

### Data collection tools and procedures

Relevant information related to associated factors of indoor air microbial load, like environmental factors (mechanical ventilation, open windows and doors, and room temperature), activities (bed making, room traffic, and room cleaning procedure), crowdedness (room size and the number of occupants per room), cleanliness (of room, bedding/linen, presence of waste and damps/wet materials and improper storage of food and drug items inside the room) were collected from observational checklists. Unique identification numbers were given to selected rooms as done in the case of Petri dishes.

### Sampling sites and air sample collection

Samples were collected from the following wards/rooms: surgical, pediatric, intensive care unit, medical, operation room, orthopedic, and gynecology. Air samples were taken by the passive (settle plate) sampling technique using Petri dishes containing a 5% sheep blood agar plate (SBA) of 9 cm diameter and were used for bacterial cultivation whereas Sabouraud dextrose agar (SDA) was employed for fungal cultivation, from each selected room of the wards. To have an appropriate surface density for counting, plates were placed one meter above the floor level and away from the doors and windows to minimize bacterial dilution for an hour [16]. This method allows bacteria or fungi in the air to settle on the respective culture media. Proper precautions were taken to prevent self-contamination by wearing personnel protective equipment. The sampling was done twice a day by taking into consideration the variations in density of occupants and environmental factors; mornings (8.00–9.00 am), and evenings (4.00–5.00 pm), maintaining an interval of two weeks [17]. Subsequently, the plates were transported to the Microbiology and Parasitology Laboratory, Department of Medical Laboratory Science. The SBA plates were then incubated aerobically at 37°C for 18–24 hours and SDA plates were kept at room temperature for 5–7 days. Parallelly, unused plates, one each for fungi and bacteria were kept as controls during the collection period [18].

### Microbial load count (Quantitative analysis)

After a specific period of incubation, fungi and bacteria were enumerated and converted to colony-forming units and expressed in terms of CFU/m^3^ by using the following formula N = 5a*10^4^ (bt) −1, where N = microbial CFU/m^3^ of indoor air; a = number of colonies per Petri dish; b = surface area of Petri dishes used (63.59 cm^2^); and t = exposure time (60 minutes), based on viable colony counts [19, 20]. The results were interpreted according to the standards of the WHO expert group for biological agents in indoor air environments [21] and also as per the European Commission Sanitary Standards for Non-industrial premises [13].

### Identifications of bacteria and fungi (Qualitative analysis)

Identification of various predominant aerobic Gram-positive and Gram-negative bacterial isolates was done as per the standard microbiological procedures [22]. Moist bacteria-like colonies were identified by Gram staining to confirm the presence of yeast-like fungi such as candida. If yeast-like colonies were confirmed, a germ tube test was done to identify the presence of *C*. *albicans*. Fungal colonies were identified based on the rate of growth, the general topography of the colony (flat, heaped, folded regularly or irregularly), colony texture (moist, glabrous, powdery, granular, velvety, cottony), and pigmentation on the surface and reverse side. Filamentous fungi were microscopically identified by lactophenol cotton blue staining [23].

### Antimicrobial susceptibility testing

The antimicrobial susceptibility testing was done on Mueller-Hinton agar (MHA) (Oxoid, UK) for each bacterial isolate by Kirby-Bauer disk diffusion method as per the CLSI guidelines. For Gram-positive bacteria, antibiotics such as penicillin (P) (10μg), cefoxitin (FOX) (30μg), chloramphenicol (CHL) (30μg), tetracycline (TC) (30μg), doxycycline (DOX) (30μg), vancomycin (VAN) (30μg), erythromycin (ERY) (15μg), gentamicin (CN) (10μg), and ciprofloxacin (CIP) (5μg) were used. For Gram-negative bacteria, ampicillin (AMP) (10μg), piperacillin (PIP) (100μg), ceftriaxone (CRO) (30μg), cefopime (CFP) (30μg), amoxicillin-clavulanate (AUG) (20μg), gentamicin (CN) (10μg), tetracycline (TTC) (30μg), chloramphenicol (CHL) (30μg), ciprofloxacin (CIP) (5μg), and meropenem (MEM) (10μg) were employed. Antibiotics were selected as per the CLSI guidelines, 2019 [24]. The presence of MRSA, ESBL, and carbapenem-resistant bacteria was detected as per the standard procedures. Multi-drug resistance in this study was extrapolated as the resistance of at least three or more groups of antibiotics tested [25].

### Data management and quality control

For data collection, an initial discussion with data collectors was organized and further training was given to them at Sikela Health Center, Arba Minch. Pre-tests were also done for both air sampling and the checklist. The purpose of the pre-test for air sampling was to examine the count of bacterial and fungal colonies during the initial one hour of the indoor media exposure (hospital rooms). A total of ten samples were included in the pre-test of air sampling and were used to check the validity of the checklist and the consistency of results to the objective of the study, which in turn assured the familiarity of data collectors. A total of ten checklists were there in the pre-test of the observational study. The reliability of findings was guaranteed by implementing quality control measures throughout the whole process of laboratory work and all materials, equipment, and procedures were adequately controlled. During air sampling, sterile gloves, masks, and protective gowns were worn to prevent the self-contamination of 5% SBA and SDA. All culture media were prepared according to the directions of manufacturers and were tested for sterility and performance. Pre-analytical, analytical and post-analytical stages of quality assurance as given in standard operating procedures of the microbiology laboratory of Ethiopian Public Institution were strictly followed. The control strains of Standard American Type Culture Collection (ATCC), such as *S*. *aureus* (ATCC 25923), *E*. *coli* (ATCC 25922), and *S*. *agalactiae* isolates (ATCC 12386) were selected to check the quality of the culture media and antimicrobial disks.

### Data analysis

Data were checked, cleaned, and coded for their completeness and entered by Epi-Data version 4.4.3.1 and exported to Statistical Package for Social Sciences (SPSS) version 25 for further analysis. One way ANOVA test was conducted to obtain the mean bacterial and fungal concentrations in the air samples of each ward. A logistic regression model was used for both bivariable & multivariable analyses to determine the association among independent variables and the indoor air microbial load grouped as per the WHO standards on indoor air (<1000 CFU/m^3^ and >1000 CFU/m^3^). Initially, the data were subjected to a series of bivariable analyses, and those variables at a cut-off point of p-value less than 0.25 were chosen for multivariable analysis. The fitness of the model was checked by the Hosmer-Lemeshow goodness fit test. Adjusted odds ratio (AOR) and 95% confidence interval (CI) were used to determine the strength of association; a p-value <0.05 in the multivariable analysis was considered statistically significant.

### Ethical clearance

The study was approved by the Institutional Research Ethics Review Board of the College of Medicine and Health Science of Arba Minch University (IRB 1043/21 dated 29-01-2021).

## Results

### Indoor air microbial load

In the present study, quantitative analyses of bacterial and fungal isolates from the indoor air of different wards in AMGH were done by the settle plate technique (passive method). The mean bacterial and fungal loads in different wards varied widely. The total average of the mean bacterial loads in selected wards was found to be 1914±1081.4 CFU/m^3^ (95% CI: 1718.5–2109.48 CFU/m^3,^). The highest mean bacterial load was found in the male surgical (2957.5±669.76CFU/m^3^) and gynecology wards (2928.33±645.6 CFU/m^3^), and the second-highest load corresponding to the female surgical ward (2515±1153 CFU/m^3^). At the same time, the least bacterial load was observed in the operation room (676.5±202.7 CFU/m^3^) (Table 1).

**Table 1:** Average means of microbial load of different wards in the AMGH versus the WHO expert group and European commission sanitary standards for non-industrial premises.

Wards	Bacteria (Mean CFU/m^3^ ± SD) n = 12	95% CI	Fungi (Mean CFU/m^3^ ± SD) n = 12	95% CI	WHO standard	European Standard
OR	676.5±202.7	547.7–805.3	632±147	538.9–7725.7	Acceptable	High
ORTHW	887.08±498.75	570.2–1204	699±454.54	410.4–988	Acceptable	High
MSW	2957.5±669.76	2531.9–3383	1972±774	1480–2464	Unacceptable	Very high
FSW	2515±1152.8	1782.5–3247.4	1787.2±915	1205–2368	Unacceptable	Very high
ICU	1095.75±351.3	872.5–1319	1053.9±367	820–1287.3	Unacceptable	High
PW	2008.5±789.73	1506.7–2510.3	1770.5±800	1261–2279.3	Unacceptable	Very high
FMW	2495±832.69	1966.2–3024.3	2288±718.6	1831–2745.2	Unacceptable	Very high
MMW	2504.42±611.7	2115.7–2893.0	2042±761	1558–2525.6	Unacceptable	Very high
E-WARD	1071.6±751.5	594.2–1549.17	953.5±547.	605–1301.5	Unacceptable	High
GYNW	2928.33±645.6	2518.08–3338	2137±484.6	1829–2444.9	Unacceptable	Very high
Total (average)	1914±1081.4	1718.52–2109	1533.7±858	1378.5–1688	Unacceptable	High

OR: Operation Room, ORTHW: Orthopedic Ward, MSW: Male surgical ward, FSW: Female surgical ward, ICU: Intensive Care Unit, PW: Pediatrics ward, FMW: Female Medical Ward, MMW: Male Medical Ward, GYNW: Gynecology ward, SD: standard deviation, n = 12 plates per room.

WHO expert group microbial load standard: Both bacterial and fungal load <1000 CFU/m^3^ as extrapolated as acceptable and either bacterial or fungal load ≥1000 CFU/m^3^ are considered as unacceptable. European Commission for non-industrial premises sanitary standard for microbial load less than 50 CFU/m^3^ as ‘very low’ bacterial load, 50–100 CFU/m^3^ as ‘low’, 100–500 CFU/m^3^ as ‘intermediate’, 500–2000 CFU/m^3^ as ‘ high’ and above 2000 CFU/m^3^ as ‘very high’.

The total average (mean) of fungal load in selected wards was 1533.7±858 CFU/ m^3^ (95% CI: 1378.5–1688 CFU/m^3^). The highest mean fungal load was found in the female medical ward (2288±718 CFU/m3), followed by gynecology (2137±484.6 CFU/m^3^) and the male surgical wards (1972±774 CFU/m^3^). The least fungal load was found in the operation room (632±147 CFU/m^3^) (Table 2). Based on the mean microbial load (comprising bacteria and fungi), male surgical and gynecology wards can be classified as highly contaminated. Interestingly, the least contaminated was the operation room, concerning the value of the microbial load (Table 1).

**Table 2:** Profiles of bacterial isolates identified from the indoor air of selected wards of AMGH, February-April 2021.

Bacterial isolates	Selected wards of AMGH										
	Frequency	Pediatrics Ward	Gynecology Ward	Orthopedic Ward	Male surgical Ward	Female surgical Ward	ICU	OR	FMW	MMW	E-ward
	n (%)										
Gram-positive	130(56.8)	24(18.5)	20(15.4)	10(7.7)	16(12.3)	11(8.5)	10(7.7)	8(6.2)	9(7)	11(8.5)	11(8.5)
*S*. *aureus*	46(20)	9(19.6)	8(17.4)	4(8.7)	7(15)	2(4.39)	3(6.5)	3(6.5)	3(6.5)	4(8.7)	3(6.5)
CoNS	40(17.5)	10(25)	5(12.5)	3(7.5)	5(12.5)	4(10)	3(7.50)	3(7.5)	2(5)	3(7.5)	2(5)
*Enterococcus* sp.	22(9.6)	3(13.4)	1(4.5)	3(13.4)	3(13.4)	3(13.4)	2(13.4)	2(18)	2(9)	1(4.5)	2(9)
*Streptococcus* sp.	11(4.8)	1(9)	2(18)	0(0)	1(9)	2(18)	1(9)	0(0)	1(9)	2(18)	1(9)
*Bacillus* sp.	7(3.1)	1(14.3)	2(28.6)	0(0)	0(0)	0(0)	0(0)	0(0)	1(14.3)	1(14.3)	2(14.3)
*Micrococcus* sp.	4(1.7)	0(0)	2(650)	0(0)	0(0)	0(0)	1(25)	0(0)	0(0)	0(0)	1(25)
Gram-negative	99(43)	21(21)	12(12)	7(7)	15(15)	11(11)	5(5)	3(3)	12(12)	8(8)	5(5)
*K*. *pneumoniae*	21(9.2)	4(19)	2(9.5)	2(9.5)	4(19)	2(9.5)	1(4.8)	1(4.8)	3(14.3)	1(4.8)	1(4.8)
*P*. *aeruginosa*	18(7.8)	4(22.2)	3(16.7)	1(5.6)	2(11)	3(16.7)	1(5.6)	0(0)	2(11)	2(11)	0(0)
*Acinetobacter* sp.	16(7)	3(18.7)	2(12.5)	1(6.3)	3(18.7)	2(12.5)	1(6.3)	1(6.3)	2(12.5)	1(6.3)	0(0)
*E*. *coli*	15(6.6)	2(13.3)	2(13.3)	0(0)	3(20)	2(13.3)	0(0)	1(6.7)	2(13.3)	1(6.7)	2(13.3)
*E*. *aerogenes*	11(4.8)	3(27.3)	1(9)	1(9)	0(0)	0(0)	2(18)	0(0)	2(18)	1(9)	1(9)
*C*. *freundii*	8(3.5)	2(25)	1(12.5)	0(0)	2(25)	1(12.5)	0(0)	0(0)	1(12.5)	1(12.5)	0(0)
*P*. *mirabilis*	6(2.6)	1(16.7)	1(16.7)	1(16.7)	1(16.7)	1(16.7)	0(0)	0(0)	0(0)	0(0)	1(16.7)
*P*. *vulgaris*	4(1.7)	2(50)	0(0)	1(25)	0(0)	0(0)	0(0)	0(0)	0(0)	1(25)	0(0)
**Total**	**229**(100)	**45**(19.6)	**32(14)**	**17**(7.4)	**31**(13.5)	**22**(9.6)	**15**(6.6)	**11**(4.8)	**21**(9.2)	**19**(8.3)	**16**(7)

ICU: Intensive Care Unit, OR: Operation Room, FMW: female medical ward, MMW: male medical ward, n: number of isolates

Statistical analysis showed that both bacterial and fungal loads in all wards differed significantly from each other with a p-value of 0.001 and 0.043 respectively. These can be correlated to the variations in the density of occupants in rooms as well as to the fluctuations in environmental factors around the rooms. While comparing the mean of microbial loads at different sampling times, it was found that values corresponding to morning and afternoon were 1827.85 and 2000.15 CFU/m^3^ respectively, and are not that significant statistically (p-value = 0.385). Likewise, the mean of fungal loads were 1420.3 and 1647 CFU/m^3^ respectively during morning and afternoon, and are also not that significant (p-value = 0.149).

The male and female surgical, female medical, male medical, pediatric, E-ward, and gynecology wards of AMGH were found to be contaminated to an unacceptable level, ie., microbial load >1000 CFU/m^3^ as per the standards of the WHO expert group. At the same time, the operation room and orthopedic ward can be considered to maintain an acceptable level of contamination, ie., microbial load <1000 CFU/m^3^ (676.5±202.7 and 887.08±498.75 CFU/m^3^ respectively) (Table 1).

Based on the sanitary standards assigned to non-industrial premises (as per the European Commission classification), the indoor air quality in wards such as OR, orthopedic, pediatric, ICU and E-wards are considered as ‘high’ (i.e. 500–2000 CFU/m^3^) in terms of both fungi and bacteria. Alarmingly, five wards such as FMW, MMW, pediatric, male surgical, and gynecology were found to have very “high” (ie., >2000 CFU/m^3^) microbial load (Table 1).

### Types and prevalence of bacterial isolates

The qualitative microbiological analyses of indoor air from ten different rooms in seven wards of AMGH are presented in Tables 2 & 3. From the total of 240 indoor air sample plates tested, 229 bacterial and 139 fungal isolates were detected, with a preponderance of Gram-positives, 130 (56.7%). Gram-positive bacterial isolates include *S*. *aureus*, CoNs, *Enterococcus* sp., *Streptococcus* sp., *Bacillus* sp., and *Micrococcus* sp. Gram-negative isolates comprised *K*. *pneumoniae*, *P*. *aeruginosa*, *Acinetobacter* sp., *E*. *aerogenes*, *Citrobacter* sp., *P*. *mirabilis*, and *P*. *vulgaris*. *Staphylococcus aureus* was the prominent isolate among the Gram-positive bacteria, accounting for 46/229 (20.1%), followed by CoNS, 40/229 (17.5%), and *Enterococcus* sp., 22/229 (9.6%). The predominant Gram-negative bacteria were isolates of *K*. *pneumoniae*, 21/229(9.2%), followed by *P*. *aeruginosa*, 18/229(7.9%), *Acinetobacter* sp., 16/229(7%), and *E*. *coli*, 15/229(6.5%).

**Table 3:** Profiles of fungal isolates identified from indoor air of selected wards of AMGH, February-April 2021.

Fungal Isolates	Frequency (percent)	Pediatrics ward	Gynecology ward	Orthopedic ward	Male surgical Ward	Female surgical Ward	ICU	OR	FMW	MMW	E-ward
	n (%)										
*Aspergillus* sp.	53(3.1)	8(15)	7(13)	2(3.8)	8(15)	7(13)	5(9.4)	2(3.8)	6(11.3)	5(9.4)	3(5.7)
*Penicillium* sp.	42(30.2)	5(12)	9(12.4)	1(2.4)	6(14.3)	4(9.5)	3(7.1)	1(2.4)	5(12)	6(14.3)	2(4.8)
*C*. *albicans*	28(20.1)	4(14.3)	2(7.1)	2(7.1)	6(21)	3(10.7)	0(0)	0(0)	6(21)	4(14.4)	1(3.6)
*Rhizopus* sp.	17(12.2)	4(23.5)	5(29.4)	0(0)	1(5.9)	4(5.9)	0(0)	0(0)	2(11.8)	1(11.8)	0(0)
*Fusarium* sp.	16(11.5)	5(31.3)	3(18.8)	0(0)	3(31.3)	1(6.3)	0(0)	0(0)	1(6.3)	0(0)	0(0)
*Mucor* sp.	13(9.4)	1(7.7)	3(23)	1(7.7)	4(30.8)	0(0)	0(0)	1(7.7)	2(15.4)	0(0)	0(0)
Total	139(100)	27(19.4)	29(20.8)	6(4.3)	28(20)	19(13.7)	8(5.8)	4(2.9)	22(15.8)	16(11.5)	6(4.3)

ICU: Intensive Care Unit, OR: Operation Room, FMW: female medical ward, MMW: male medical ward

### Types and prevalence of fungal isolates

The fungal isolates detected in our study include *Aspergillus* sp., *Penicillium* sp., *Fusarium* sp., *C*. *albicans*, *Rhizopus* sp., and *Mucor* sp. The most predominant fungal isolates were *Aspergillus* sp. corresponding to 53/139(38%), followed by *Penicillium* sp., 42/139 (30.2%) and *C*. *albicans*, 28/139 (20.1%) (Table 3).

### Ward-wise distribution of bacterial and fungal isolates

Results revealed that among all the wards studied, pediatric, 45/229(19.6%), followed by gynecology, 32/229(14%), and male surgical, 31/229(13.5%) were highly contaminated with both Gram-positive and Gram-negative bacteria. The least bacterial contaminated ward was the operation room 11/229(4.8%). Pediatric, 24/130(18.5%), gynecology, 20/130(15.4%), and male surgical, 16/130(12.3%) wards contain the highest number of Gram-positive bacteria. The majority of isolates of *S*. *aureus* were distributed in three wards such as pediatric, 9/46(19.6%), gynecology, 8/46(17.4%), and male surgical, 7/46(15.2%). The second most common Gram-positive isolates, CoNs was mainly distributed in the pediatric, 10/40(25%), gynecology, 5/40(12.5%), and male surgical wards, 5/40(12.5%). Results revealed that the pediatric ward, 21/99(21%), MSW, 15/99(15%), FMW, 12/99(12%), and gynecology wards, 12/99(12%) were contaminated with Gram-negative bacteria. Isolates of *K*. *pneumoniae*, *P*. *aeruginosa* (except OR and E-ward), and *Acinetobacter* sp. (except E-ward) were more or less equally distributed in all the wards.

The overall results revealed a predominance of fungal isolates in three wards such as the gynecology, 27/139 (19.4), MSW, 29/139(21%), and pediatric, 28/139(20%), and the least fungal contaminated ward was the operation room, 4/139(2.9%). The major isolate of *Aspergillus* sp. was mainly distributed in the pediatric ward, 8/53(15%), MSW, 8/53(15%), gynecology ward, 7/53(13%), FSW, 7/53(13%), and FMW, 6/53(11%). The second most common isolate, *Penicillium* spp. was mainly retrieved from the gynecology ward, 9/42(21.5), MSW, 6/42(14.3%), and MMW, 6/42(14.3%); *C*. *albicans* was the frequently obtained type of yeast isolate from MSW, 6/28(21.5%) and FMW, 6/24 (21.5%).

### Antimicrobial susceptibility profiles of Gram-positive isolates

Gram-positive isolates showed wider variations in their susceptibility profiles. Most of these isolates showed resistance against ceftriaxone, penicillin, tetracycline, erythromycin, ie., 16/22 (73%), 70/115(61%), 75/130(58%), and 67/119(56%) respectively. Remarkably, higher percentages of susceptibilities were produced by these isolates against a series of antibiotics such as chloramphenicol, 108/130(83%), clindamycin, 68/130(70%), and cefoxitin, 60/90(67%) (Table 4).

**Table 4:** Antibiotic susceptibility profiles of Gram-positive bacteria isolated from indoor air of selected wards of AMGH, February-April 2021.

Bacterial Isolate	Susceptibility Pattern n (%)	Antimicrobial agents													
		CTR	CFP	GEN	TTC	CHL	CIP	MER	PEN	CXT	DOX	VAN	ERY	SXT	CLN
*S*. *aureus*n = 46	S	-	-	26(57)	31(67)	43(93)	31(67)	-	21(46)	26(57)	39 (85)	-	23 (50)	30(65)	34 (74)
	R	-	-	20(43)	15(33)	3(7)	15(33)	-	25(54)	20(43)	7(15)	-	23 (50)	16(35)	12 (26)
CoNS n = 40	S	-	-	23(58)	12(30)	35(88)	30(75)	-	24(60)	32(80)	22 (55)	-	17 (42)	11(27)	27 (67)
	R	-	-	17(42)	28(70)	5(12)	10(25)	-	16(40)	8(20)	18 (45)	-	23(58)	29(73)	13 (33)
*Enterococcus* sp. n = 22	S	-	-	-	2(9)	16(73)	4(18)	-	0(0)	-	2(9)	4 (18)	4(18)	-	-
	R	-	-	-	20(91)	6(27)	18(82)	-	22(100%)	-	20(91)	18(82)	18(82)	-	-
*Streptococcus* sp. n = 11	S	3(27)	10(91)	-	6(55)	9(82)	-	11(100)	-	-	-	-	8(73)	-	7 (64)
	R	8(73)	1(9)	-	5(45)	2(18)	-	0(0)	-	-	-	-	3(27)	-	4 (37)
*Bacillus* sp. n = 7	S	2(29)	1(14)	0(0)	3(43)	4(57)	0(0)	2(29)	0(0)	-	3(43)	-	-	-	-
	R	5(71)	6(86)	7(100)	4(57)	3(43)	7(100)	5(71)	7(100)	-	4 (57)	-	-	-	-
*Micrococcus* sp. = 4	S	1(25)	0(0)	2(50)	1(25)	1(25)	0(0)	0(0)	-	2 (50)	0 (0)	-	-	-	-
	R	3(75)	4(100)	2(50)	3(75)	3(75)	4(100)	4(100)	-	2 (50)	4 (100)	-	-	-	-
Total tested		22	22	97	130	130	119	22	115	90	119	22	119	86	97
Average S	S	6(27)	11(50)	51(53)	55(42)	108(83)	65(55)	13(59)	45(39)	60(67)	66 (56)	4(18)	52(44)	42 (48)	68 (70)
Average R	R	16(73)	11(50)	46(47)	75(58)	22(17)	54(45)	9(41)	70(61)	30(33)	53 (44)	18(82)	67(56)	45 (52)	29 (30)

CTR: ceftriaxone, CFP: cefopime, GEN: gentamicin, TTC: tetracycline, CHL: chloramphenicol, CIP: ciprofloxacin, MER: meropenem, CXT: cefoxitin, DOX: doxycycline, VAN: vancomycin, ERY: erythromycin, SXT: sulfamethoxazole-trimethoprim, CLN: clindamycin.

The dominant isolate, *S*. *aureus* showed a varied extent of resistance to penicillin, 25/46(54%), erythromycin, 23/46(50%), gentamicin, and cefoxitin, each of which corresponded to 20/46(43%) to each. However, comparatively lower resistances only were produced against the antibiotics, trimethoprim-sulfamethoxazole, 16/46(35%), tetracycline, ciprofloxacin, 15/46(33%), and clindamycin, 12/46(26%). Chloramphenicol, 43/46(93%) and doxycycline, 39/46(85%) were found to be very effective against *S*. *aureus*. Among the 46 isolates of *S*. *aureus*, 18 showed a zone of inhibition ≤21mm (16.2 to 19.5 mm), in the cefoxitin disk diffusion assay and were considered methicillin-resistant *S*. *aureus* and the percentage of MRSA among *S*. *aureus* was 20/46 (43%). Isolates of CoNs showed higher resistance to trimethoprim-sulfamethoxazole, 29/40(73%), tetracycline, 28/40(70%), and erythromycin, 23/40(58%). Only lower resistances were produced against doxycycline, 18/40(45%), gentamicin, 17/40(42%), penicillin, 16/40(40%), clindamycin, 13/40(33%), and ciprofloxacin, 9/40(25%). Chloramphenicol, 35/49(88%) and cefoxitin 32/40(80%) were also found to be effective. Isolates of *Enterococcus* sp. were resistant, 22/22(100%) to penicillin, 20/22(91%) each to tetracycline and doxycycline, and 18/22(82%) to other three drugs tested (ciprofloxacin, vancomycin, and erythromycin). Chloramphenicol was the only effective drug, 16/22 (73%) against *Enterococcus* sp. (Table 4).

Isolates of *Streptococcus* sp. were much resistant 8/11(73%) to ceftriaxone, but lower resistance only was produced by them against tetracycline, 5/11(45%) and clindamycin, 4/11(37%). At the same time, 11/11(100), 10/11(91), 9/11(82), and 8/11(73%) isolates of *Streptococcus* sp. showed susceptibility towards meropenem, cefopime, chloramphenicol, and erythromycin respectively. Isolates of *Bacillus* sp. showed resistance to five of the tested drugs (ie., 71–100%), but exhibited only moderate to lower resistance to each of doxycycline and meropenem, 4/7(57%), and chloramphenicol, 3/7(43%). Isolates of *Micrococcus* sp. showed a wider variation in resistance, in the range of 50–100% against all the tested drugs (Table 4).

### Antimicrobial susceptibility profiles of Gram-negative isolates

Susceptibility profiles of Gram-negative bacteria (n = 99) isolated from the indoor air of AMGH wards, against eleven antibiotics are presented in Table 5. The extent of antimicrobial resistance shown by Gram-negative organisms corresponded to a wider range, ie., from 35 to 75%. These organisms exhibited considerable resistance ie., 61/81 (75%), 53/77 (69%), 31/46(67%), and 63/99 respectively against trimethoprim-sulfamethoxazole, tetracycline, amoxicillin-clavulanate, and meropenem. At the same time, a higher extent of susceptibility was produced against antibiotics such as chloramphenicol, 40/65(62%), piperacillin, 65/99(65%), and ceftriaxone, 46/81(57%).

**Table 5:** Antibiotic susceptibility profiles of Gram-negative bacteria isolated from indoor air of selected wards of AMGH, February-April 2021.

Bacterial Isolate	Susceptibility Pattern n(%)	Antimicrobial agents										
		AMP	PIP	CTR	CFP	AGU	GEN	CHL	CIP	MER	TTC	SXT
*E*. *coli* n = 15	S	9(60)	13(87)	8(53)	5(33)	5(33)	9(60)	13(87)	8(53)	6(40)	3(20)	4(40)
	R	6(40)	2(13)	7(47)	10(67)	10(67)	6(40)	2(13)	7(47)	9(60)	12(80)	9(60)
*K*. *pneumoniae* n = 21	S	-	18(86)	13(62)	13(62)	7(33)	7(34)	8(38)	3(14)	8(38)	4(19)	6(29)
	R	-	3(14)	8(38)	8(38)	14(67)	14(66)	13(62)	18(86)	13(62)	17(81)	15(71)
*E*. *aerogenes* n = 11	S	-	7(64)	7(64)	5(45)	-	6(55)	9(82)	7(63)	4(37)	2(18)	3(28)
	R	-	4(36)	4(36)	6(55)	-	5(45)	2(18)	4(37)	7(63)	9(82)	8(72)
*C*. *freundii* n = 8	S	-	5(62)	7(88)	6(75)	-	4(50)	7(88)	3(38)	4(50)	5(62)	1(13)
	R	-	3(38)	1(12)	2(25)	-	4(50)	1(12)	5(62)	4(50)	3(38)	7(87)
*P*. *vulgaris* n = 4	S	-	2(50)	3(75)	2(50)	2(50)	0(0)	1(25)	2(50)	2(50)	-	0(0)
	R	-	2(50)	1(25)	2(50)	2(50)	4(100)	3(75)	2(50)	2(50)	-	4(100)
*P*. *mirabilis* n = 6	S	4(67)	5(83)	3(50)	4(67)	1(17)	3(50)	2(33)	3(50)	2(33)	1(17)	2(33)
	R	2(33)	1(17)	3(50)	2(33)	5(83)	3(50)	4(67)	3(50)	4(67)	5(83)	4(67)
*P*. *aeruginosa* n = 18	S	-	5(28)	-	6(33)	-	6(33)	-	7(39)	5(28)	-	-
	R	-	13(72)	-	12(67)	-	12(67)	-	11(61)	13(72)	-	-
*Acinetobacter* sp. n = 16	S	-	10(62)	5(31)	8(50)	-	8(50)	-	5(31)	6(38)	9(56)	4(25)
	R	-	6(38)	11(69)	8(50)	-	8(50)	-	11(69)	10(62)	7(44)	12(75)
Total tested isolates		22	99	81	99	46	99	65	99	99	77	81
Average susceptibility		13(59)	65(65)	46(57)	49(50)	15(33)	43(43)	40(62)	38(38)	37(37)	24(31)	20(25
Average resistant		9(41)	34(35)	35(43)	50(50)	31(67)	56(56)	25(38)	62(62)	63(63)	53(69)	61(75)

AMP: ampicillin, PIP: piperacillin, CFP: cefopime, CTR: ceftriaxone, GEN: gentamicin, CIP: ciprofloxacin, MER: meropenem, CHL: chloramphenicol, TTC: tetracycline, SXT: sulfamethoxazole-trimethoprim, AGU: amoxicillin-clavulanate, R: resistant, S: susceptible

The predominant isolate, *K*. *pneumoniae* demonstrated a higher level of resistance against ciprofloxacin, 18/21(86%), tetracycline, 17/21(81%), trimethoprim-sulfamethoxazole, 15/21(71%), amoxicillin-clavulanate, 14/21(67%) and gentamicin, 14/21(67%) (Table 5). *Piperacillin* was relatively effective against most of the isolates of *K*. *pneumoniae* corresponding to the susceptibility of 18/21(86%). Isolates of *P*. *aeruginosa* were found to be fairly resistant to all the five tested antibiotics ie., in the range of 61 to 72% (Table 5). Isolates of *Acinetobacter* sp. also were found to be resistant to trimethoprim-sulfamethoxazole, ie., 12/16(75%); ceftriaxone and ciprofloxacin, each 11/16(69%) and also meropenem, 10/16(62%). On the other hand, 10/16(62%) and 9/16(56%) of their isolates were found to be susceptible to piperacillin and tetracycline respectively.

Isolates of *E*. *coli* were resistant to tetracycline, ie., 12/15(80%), whereas 10/15(67%) of them were resistant, each to cefopime and amoxicillin-clavulanate; these isolates also showed the same level of resistance, ie., 9/15(60%) to two drugs tested, ie., meropenem and trimethoprim-sulfamethoxazole. Comparatively, isolates of *E*. *coli* showed only a medium level of resistance, ie., 8/15(53.3%) against each ceftriaxone and ciprofloxacin, and an equal level of resistance, ie., 6/15(40%) in the case of ampicillin and gentamicin. The high susceptibility, ie., 13/15 (87%) shown by the isolates of *E*. *coli* to both *piperacillin* and chloramphenicol is notable. Isolates of *E*. *aerogenes* were resistant against tetracycline, trimethoprim-sulfamethoxazole, and meropenem, ie., 9/11(82%), 8/11(72%), and 7/11(63%) respectively. The same extent of resistance was produced against cefopime, ie., 6/11(55%). Chloramphenicol was an effective drug, 9/11(82%) against *Enterobacter* isolates.

Isolates of *C*. *freundii* were resistant to the most commonly prescribed antibiotics such as trimethoprim-sulfamethoxazole, 7/8(87.5%), and ciprofloxacin, 5/8(62%). Notably, the isolates of the same species were equally susceptible to both chloramphenicol and ceftriaxone, ie., 7/8(88%) and 6/8(75%) to cefopime (Table 5).

In the case of *Proteus*, isolates of *P*. *vulgaris* were resistant, 4/4(100%) against two drugs tested (gentamicin, and trimethoprim-sulfamethoxazole), and 3/4(75%) were resistant to chloramphenicol. Furthermore, these isolates produced 2/4(50%) resistance to five drugs tested, ie., meropenem, ciprofloxacin, amoxicillin-clavulanate, cefopime, and *piperacillin*. Interestingly, 3/4(75%) of them showed susceptibility to ceftriaxone, whereas, 5/6(83%) of the isolates of *P*. *mirabilis* produced resistance against both tetracycline and amoxicillin-clavulanate. Besides, 4/6(67%) of the isolates of *P*. *mirabilis* showed resistance to three drugs, ie., trimethoprim-sulfamethoxazole, meropenem, and chloramphenicol. At the same time, resistance exhibited against three drugs such as gentamicin, ciprofloxacin, and ceftriaxone was 3/6(50%) only. It is noted that 5/6(83%) of these isolates were susceptible to *piperacillin*, whereas the susceptibility to ampicillin and cefopime each was the same, 4/6(67%) only (Table 5).

Altogether, it is clear that the isolates of Gram-negative bacilli were highly resistant to trimethoprim-sulfamethoxazole (75%) and tetracycline (69%); however, they were only moderately susceptible to *piperacillin (65%)* and chloramphenicol (62%), excluding the isolates of *P*. *aeruginosa*.

### MDR profiles of bacterial isolates

Out of the 229 total bacterial isolates, 122 were found to be MDR (53.2%) of which, 54/99(54.5%) belong to the Gram-negative group and 68/130(52.3%) were Gram-positives. The MDR Gram-positive bacteria consist of 26/46(56.5%) of *S*. *aureus*, 21/40(52.5%) CoNs, 8/22(36.4%) *Enterococci* sp., 7/7(100%), *Streptococcus* sp. 2/11 (18.2%) and 4/4(100%) each of *Bacillus* sp. and *Micrococcus* sp. respectively. Among the Gram-negative bacteria, MDR types comprise 7/15 (46.6%) of *E*. *coli*, *P*. *vulgaris*, 1/4 (25%), *C*. *freundii*, 4/8 (50%), *P*. *aeruginosa*, 8/18(44.4%), *Acinetobacter* sp., 12/16(75%) and *K*. *pneumoniae*, 12/21(57%) (Table 6).

**Table 6:** MDR, ESBLs and carbapenemase enzyme production patterns of Gram-negative isolates.

Bacterial isolates	MDR n (%)	ESBLs producers n (%)	Carbapenemase producers n (%)	ESBLs and carbapenemase producers n (%)
*E*. *coli* (n = 15)	7(46.6)	5(33.3)	3(20)	2(13.3)
*K*. *pneumoniae* (n = 21)	12(57)	6(28.5)	5(23.8)	3(14.3)
*E*. *aerogenes aerogenes* (n = 11)	6(54.5)	2(18.2)	1(9)	0(0)
*P*. *mirabilis* (n = 6)	4(66.7)	2(33.3)	1(16.7)	0(0)
*P*. *aeruginosa* (n = 18)	8(44.4)	3(16.7)	13(72.2)	1(5.5)
*Acinetobacter* sp. (n = 16)	12(75)	7(43.8)	2(12.5)	0(0)
*C*. *freundii* (n = 8)	4(50)	1(12.5)	0(0)	0(0)
*P*. *vulgaris* (n = 4)	1(25)	0(0)	0(0)	0(0)
Gram-negative Total (n = 99)	54(54)	26(26.3)	25(25)	6(6)
*S*. *aureus* (n = 46)	26(56.5)	ND	ND	ND
CoNS (n = 40)	21(52.5)	ND	ND	ND
*Enterococcus* sp.(n = 22)	8(36.4)	ND	ND	ND
*Streptococcus* sp. (n = 11)	2(18.2)	ND	ND	ND
*Bacillus* sp. (n = 7)	7(100)	ND	ND	ND
*Micrococcus* sp. (n = 4)	4(100)	ND	ND	ND
Gram-positive Total(n = 130)	68(52.3)	ND	ND	ND
Average total (n = 229)	**122(53.3)**	ND	ND	ND

MDR: multiple drug resistant, ESBL: extended spectrum beta-lactamase, n: isolates, ND = not done

### ESBL and carbapenemase-producing isolates of Gram-negative bacilli

Out of the 99 isolates of Gram-negative bacilli, 26/99(26.3%) were found to be ESBL producers and carbapenemase production was observed in the case of 25/99(25%). The co-existence of both ESBL and carbapenemase producers was seen in the case of 6(6%) whereas the production of ESBL was confirmed in the case of 7/16(43.8%) isolates of *Acinetobacter* sp., *E*. *coli*, 5/15(33.3%) and *P*. *mirabilis*, 2/6(33.3%). On the other hand, a high percentage of carbapenemase production was seen in the case of *P*. *aeruginosa*, 13/18(72.2%), followed by *K*. *pneumoniae*, 5/21(23.8%). Besides, the isolates of *K*. *pneumoniae* were found to be associated with a high percentage production of both ESBLS and carbapenemase (3/21; 14.3%) (Table 6).

### Factors associated with microbial load

Various factors (like crowd index of occupants in the room, sanitary conditions, availability of mechanical ventilation, and environmental factor such as temperature) were analyzed to find any probable association with indoor air microbial load in hospital wards. For statistical analysis, only WHO standards for the permissible level of indoor air microbial loads were considered. In bivariable logistic regression analysis, microbial loads in the indoor air of wards were found to be statistically significant (p<0.25), in the case of most of the variables studied (Table 7).

**Table 7:** Logistic regression model analysis of independent variables associated to the indoor air microbial load.

Characteristics	WHO expert group standard		Bivariable analysis		Multivariable analysis	
	<1000 CFU/m^3^	≥1000 CFU/m^3^	P-value	COR (95%CI)	P-value	AOR (95%CI)
**Room crowd index**	N (%)					
>2(high)	8(6.7%)	55(45.8%)	0.00*	25.8(8.8–75.6)	0.003**	12.5(2.42–65)
1-2(medium)	10(8.3%)	9(7.5%)	0.045*	3.4 (1.02–11.1)	0.425	2.14(0.33–13.8)
<1(low)	30(25%)	8(6.7%)	1	1		
**Mechanical ventilation**				1		
Yes	20(16.7%)	35(29.2%)	1			
No	28(20.8%)	37(30.8%)	0.455	0.755 (0.36–1.58)	-	-
**Room temperature**						
Below 25^0^c	21(17.5%)	25(20.8%)	1			
25-28^0^c	12(10%)	4(3.3%)	0.086*	2.551 (0.87–7.4)	0.37	2.75(0.29–25.5)
Above 28^0^c	11(9.2%)	38(31.7%)	0.00*	7.143(2.36–21.57)	0.52	1.6(0.38–6.8)
**Damp/wet material**						
Yes	5(4.2%)	30(25%)	0.001*	6.14(2.18–17.34)	0.025**	7(1.3–37.4)
No	43(35.8%)	42(35%)	1	1		
**Activity of room renovation**						
Yes	16(13.3)	22(18.3%)	0.74	0.88(0.403–1.92)	-	-
No	32(26.7%)	50(41.7%)	1	1		
**Bed making activity**						
**Yes**	10(8.3%)	26(21.2%)	0.07*	2.148 (0.921–5.0)	0.35	0.5(0.121–2.1)
**no**	38(31.7%)	46(38.3%)	1	1		
**Room traffic**						
high	16(13.3%)	60(50%)	0.001*	14.06 (4.1–48)	0.036**	9.6(1.2–79.3)
medium	17(14.2%)	8(6.7%)	0.42	1.76(0.441–7.06)	0.418	2.6(0.25–27)
low	15(12.5%)	4(3.3%)	1	1		
**Cleanliness of work area**						
Yes	29(24.2%)	33(27.5%)	1			
No	19(15.8%)	39(32.5%)	0.119*	1.8 (0.86–3.8)	0.54	1.5(0.4–5.7)
**Opened windows and doors**						
Yes	26(21.2)	35(29.2%)	1	1		
No	22(18.3%)	37(30.8%)	0.551	1.25 (0.601–2.598)	-	-
**Cleanliness(unsoiled) of bedding/linen**						
Yes	26(21.7%)	23(19.2%)	1	1		
No	22(18.3%)	49(40.8%)	0.016*	2.52 (1.185–5.34)	0.56	1.4(0.43–4.7)
**Appropriate storage of food and drug items**						
Yes	22(18.3%)	59(49.2%)	1	1		
No	23(19.2%)	22(18.3%)	0.001*	5.37(2.3–12.26)	0.008**	7.5(1.7–32)
**Presence of waste materials**						
Yes	22(18.3%)	22(18.3%)	1	1		
No	25(20.8%)	50(41.2%)	0.056*	2.1(0.98–4.455)	0.43	0.58(0.15–2.23)
**Cleanliness around the room**						
Yes	31(25.8%)	7(5.8)	1	1		
No	17(14.2%)	65(54.2%)	0.001*	17(6.3–45.1)	0.03**	5.8(1.2–28)

Note:

*Statistically significant at P<0.25

** statistically significant at P<0.05, AOR: Adjusted odds ratio, COR: Crude odds ratio, 1: reference group, CI: Confidence interval

In multivariable logistic regression analyses, only five variables were found to be statistically significant (p<0.05), with respect to the indoor air microbial loads, such as the high room crowd index [p = 0.003; AOR 12.5 (CI 95%: 2.42–65)], presence of damp/wet materials inside the wards [p = 0.025; AOR 7 (CI 95%: 1.3–37.4)], high room traffic [p = 0.004; AOR 9.6 (CI 95%: 1.2–79.3)], inappropriate storage of food and drugs inside the wards [p = 0.008; AOR 7.5 (CI 95%: 1.7–32)], and unclean environment around the wards [p = 0.03; AOR 5.8 (CI 95%: 1.2–28)].

## Discussions

The microbiological quality of indoor air in hospitals can be considered a reflection of the hygienic conditions existing in the environment [26]. In this work, the mean bacterial load in the indoor air of all studied wards, OR, and ICU in AMGH taken together was found to be 1914±1081.4 CFU/m^3^ (95% CI: 1718.5–2109.4 CFU/m^3^). Our results were comparable to the outcome of a few studies, for instance, a work done in Tamale Teaching Hospital, Ghana correspond to a bacterial load ranging between 277.6–5395.1 CFU/m^3^ [9] and another study done at Gondar University Teaching Hospital in the northwestern part of Ethiopia reported an average bacterial load of 1468 CFU/m^3^ [10]. However, these are much higher than that reported from southern Thailand (an average load of 418.79 CFU/m^3^) [27], and Nigeria (3.0 CFU/m^3^ to 76.0CFU/m^3^) [28]. Likewise, another study from the southern part of Nigeria reported a bacterial load with a mean of only 80.0 CFU/m^3^ [8]. In contrast, only lower values of bacterial load were found in a couple of independent studies done in Jimma University Specialized Hospital (3106 to 9733 CFU/m^3^) [17] and Hawassa University Comprehensive Specialized Hospital (4420 CFU/m^3^), both in Ethiopia [11]. The presence of a higher load of microorganisms in indoor air represents an important source of contamination [29].

The mean fungal load observed in the present study is 1533.7±858.8 CFU/ m^3^ (95% CI: 1378.5–1688 CFU/m^3^) and is higher than the values of previous studies done in India (0 to 262 CFU/m^3^) and Nigeria (6 to 44.7CFU/m^3^) [26, 28]. The variations could be attributed to the differences in the methods of air sampling employed (active or passive), type of wards (single bed or multi-bed), restricted areas (operational room), length of plate exposure time, and the grade/ level of hospitals (general or referral) [30]. In our study, the microbial loads that were measured at different sampling times (morning and afternoon) were not significantly different from each other and are in contrast with the results of a previous study done in Jimma [17].

The highest average indoor air bacterial load in AMGH is from the male surgical and gynecological wards. Patients in surgical and gynecological wards are likely at higher risk of being infected. This is in line with the results of some earlier studies done in Nigeria [14] and also in Hawassa, Ethiopia [31]. A study conducted in Jimma, Ethiopia [17], demonstrated higher bacterial loads in the maternity ward, followed by the medical and surgical wards. At the same time, in the case of fungi, higher loads were observed in the female medical and gynecology wards. The magnitude of contamination dealt with in this study could be attributed to the inadequacy of hygiene practiced by cleaning personnel, and can be related to products, or even procedures. Also, another possible reason could be the absence of strict control of room trafficking in these wards.

An extensive literature survey indicated that there are no uniform international standards available for assessing the extent or limits of indoor air microbial loads. We have used two different international standards for comparing the indoor air microbial loads. With respect to the WHO standard, out of the ten rooms of different wards studied including ICU and OR, only two (the operation room and orthopedic ward) were found to maintain an acceptable level, ie., 1000 CFU/m^3^ [32]. A study was done in another city in Ethiopia (Gondar) reported that the mean bacteriological loads in the majority of the wards are unacceptable [10]. Invariably, the microbial loads of all the wards in our study fall under the high and very high category of sanitary standards set by the European Commission for non-industrial premises [13]. Our result is more or less similar to another study done in Gondar, Ethiopia [10]. The probable reasons for the currently found high microbial loads in the air of different wards in AMGH are lack of mechanical ventilation, inadequate size of rooms, the higher number of occupants, and low frequency of cleaning. However, further in-depth studies are required to figure out the influence of these factors accurately [33].

In this study, various types of bacterial and fungal isolates are identified from the indoor air samples collected from various wards of AMGH, and similar profiles of bacterial isolates were reported by a couple of researchers from Ethiopia [34, 35]. Among the bacterial isolates, the majority are Gram-positives, ie., 56.7% and this is comparable to the results earlier published from two hospitals in Ethiopia [34, 35]. However, contrary to our results, a study was done in another hospital in Ethiopia (Hawassa) reported that Gram-negative bacteria are the predominant isolates [31]. The higher load of Gram-positive bacteria observed in our study could be correlated to their lower susceptibility to environmental stresses, the presence of pigments, and higher peptidoglycan contents in their cell walls, which protect from excessive heat and drying.

Among the Gram-positive bacteria, *S*. *aureus*, 46(20%) was the most frequently isolated type from all the wards. These findings are in line with the results of earlier studies published in India and Ethiopia itself [26, 35]. A probable reason for the highest prevalence of *S*. *aureus* could be its widespread existence in the hospital environment as a contaminant, which might have a suspended presence in the air [36].

Isolates of CoNs were the second most frequently observed Gram-positive bacteria and the same trend was also found in a previous study conducted in Ethiopia [34]. In the case of Gram-negative bacteria, the most predominant isolates were that of *K*. *pneumoniae*, and this was in concordance with the findings from other hospitals in Ethiopia and India [26, 35]. The predominant Gram-negative non-lactose fermenting isolate was *P*. *aeruginosa* as in the cases of a series of studies done in different hospitals in Ethiopia (Woliata and Adama) and India [26, 34, 35]. The prevalence of *Acinetobacter* observed in this study is comparable to a certain extent to another work done in Ethiopia [34]. At the same time, isolates of *E*. *coli* were also observed in our study and its percentage is somewhat similar to the range reported from Ethiopia (Hawassa) [31]. The divergence in type and percentage of bacterial isolates found in various studies could be linked to the disparities associated with the prevailing standards of air hygiene and environmental sanitation practices.

Among the fungal isolates, the most commonly observed are *Aspergillus* sp., *Penicillium* sp., and *C*. *albicans*, and these observations by and large resemble those of studies conducted in Ghana, Nigeria, and Thailand [9, 27, 30]. This study showed that the dominant fungal isolates are the species of *Aspergillus;* however, this is not in line with a study done in some parts of India, according to which the prominent isolates were *Candida* sp. and *Aspergillus* sp. [26]. Nevertheless, the load and types of fungal isolates found in our study are high and broad, and hence cannot be ignored as harmless environmental contaminants, particularly with respect to the welfare of patients with compromised immunity.

One of the factors that make bacteria more viable and effective in the hospital environment is their resistance to antibiotics. It is to be noted that 54% of *S*. *aureus* are resistant to penicillin and this is similar to an earlier trend found in Hawassa, Ethiopia [31]. The findings of the current study also indicated that 43% of *S*. *aureus* are MRSA strains and this resembles a previous study done in Wolaita Sodo (39%), Ethiopia [34]. Isolates of the second most predominant Gram-positive bacteria, CoNs showed varying degrees of resistance to different antibiotics, ie., trimethoprim-sulfamethoxazole, 73%, gentamicin, 42%, and ciprofloxacin, 25%. A similar trend in resistance profile was observed in a previous study conducted in Wolaita Sodo, Ethiopia [34].

Isolates of the predominant Gram-negative bacilli, *K*. *pneumoniae* displayed the highest resistance to two antibiotics, such as ciprofloxacin and tetracycline and this profile is comparable to the results of a prior study done in Hawassa, Ethiopia [31]. Besides, susceptibility profiles exhibited by isolates against trimethoprim-sulfamethoxazole, gentamicin, and amoxicillin-clavulanate were relatively similar to the results of an earlier work done in Addis Ababa, Ethiopia [37].

The second most predominant Gram-negative bacilli, *P*. *aeruginosa* showed resistance to all the five antibiotics tested. A similar trend in resistance profile shown by *P*. *aeruginosa* was observed against only a couple of antibiotics (gentamicin and ciprofloxacin) as per the studies conducted in other hospitals in Ethiopia (Hawassa, Wolaita Sodo, and Adama) [31, 34, 35]. Isolates of *Acinetobacter* sp. showed the highest resistance to trimethoprim-sulfamethoxazole followed by ceftriaxone and ciprofloxacin, which were similar to the results already documented in a previous study (Wolaita Sodo) [34]. Bacterial isolates of *E*. *coli*, exhibited a similar trend in resistance to three antibiotics such as tetracycline, trimethoprim-sulfamethoxazole, and ciprofloxacin and it parallels the results of a recent study done in Ethiopia (Wolaita Sodo) [34].

In our study, MDR was observed in the case of 53% of isolates, which is comparatively lower than that found in a couple of studies conducted in Wolaita Sodo (75%) [34] and Hawassa (73.3%) [31]. Likewise, in the former study, 74.6% of the isolates were Gram-positive and 84% were Gram-negative bacteria which is also higher than that found in our study [34].

The overall ESBL producers in our study contribute 26%, comprising seven species of Gram-negative bacteria. The highest percentage of ESBL producers corresponds to *Acinetobacter* sp., 43% and this is comparable to the value reported from Wolaita Sodo (55.8%) [38]; at the same time, the percentage of ESBL producing *P*. *aeruginosa* was lower (16.7%) than that found in a previous study (62.8%) (Wolaita Sodo) [38]. In the current study, 25% of Gram-negative isolates were carbapenemase producers, particularly the isolates of *K*. *pneumoniae* (23.8%) and *E*. *coli* (20%). Studies pertaining to the combined presence of ESBL and carbapenemase-producing bacteria in the indoor air of hospitals are not reported elsewhere. Bacterial isolates, such as Vancomycin-Resistant Enterococci (VRE), MRSA, ESBL, and carbapenemase-producing strains that are listed as top priority pathogens by WHO are detected in our study, which is alarming. What is striking from our results is that more than half of the bacterial isolates obtained are multi-drug resistant and hence, it can be implied that these microbes are part of hospital flora. There exists a probability of nosocomial infection associated with MDR bacterial isolates in the near or distant future and interventions from the infection prevention and control team are thus the need of the hour.

Among the various associated factors analyzed, higher room crowd index, presence of damp/wet materials inside wards, severe room traffic, inappropriate storage of food and drugs in the wards, and unclean environment in and around the wards were found to be significantly associated. For instance, a room with a higher crowd index was found to be 12.5 times more prone to be contaminated with a high microbial load. In general, the higher the occupancy level in the room, the greater the microbial bio-burden in the indoor air [39]. As per our study, intense room traffic, as well as inappropriate storage of food and drug items in the wards, can raise the contamination level further by 9.6 and 7.5 folds respectively, in comparison to properly maintained rooms/wards. A previous study reported from Harar, Ethiopia [12], found that the odds of higher bacterial loads were 8.9 times higher in the case of rooms with improper storage of food and drugs. The unclean environment around the wards was found to be 5.8 times more likely to have contamination of high microbial load, compared to cleaner premises. Our results are in parity with a study done in Ethiopia earlier that, soiled working areas have a bacterial load 12.9 times higher than a cleaner one [12].

Shortcomings of our work include the type of study design (cross-sectional), and the shorter duration of sample collection. Neonatal ICU, TB wards, outpatients departments, and offices were not included in the study due to the time constraints and limited manpower. This study is a single institution based (other hospitals are not included). Only a limited number of rooms/wards were studied and the influence of seasonal variations is not incorporated. Conventional methods of isolation of microbes were only employed and fastidious microorganisms were not investigated. Pathogenicity and virulence factors of airborne microbes also were not studied. Molecular detection of virulence and antimicrobial resistance genes of the major isolates was not performed due to the lack of infrastructure/ facilities. The influence of associated factors like humidity was not analyzed. There are no CLSI guidelines available for *Bacillus* and *Micrococcus* sp., and therefore, antimicrobial susceptibility tests were done and extrapolated based on the drugs recommended to other Gram-positive bacteria.

## Conclusions

Indoor air microbial load in AMGH is at a moderate level compared to other studies done in Ethiopia. As per WHO and European Commission standards on indoor air microbial load, the majority and/or all of the wards in AMGH were found to stand high and are not at an acceptable level. The highest mean bacterial and fungal loads were found in male surgical and female medical wards respectively. Gram-positive bacteria were predominant, particularly the isolates of *S*. *aureus* and CoNS. The predominant fungal isolates were *Aspergillus* sp., *Penicillium* sp., and *C*. *albicans*. An alarming finding is that bacterial isolates, involve VRE, MRSA, ESBL, and carbapenemase-producing strains that are listed as top priority pathogens by WHO. High room crowd index, presence of damp/wet materials inside the wards, intense room traffic, inappropriate storage of food and drug items inside the rooms, and unclean environment in and around the wards were the associated factors related to the existence of higher microbial load. Therefore, periodic air surveillance and infection prevention control program are required to minimize the transmission of these microbes to inpatients, by-standers, and healthcare workers.

## Supporting information

S1 File Supplementary file for the bacterial isolates and antimicrobial resistance profile. (XLSX)

S2 File Supplementary file for the associated factors of microbial load compared to WHO expert group standards. (XLSX)
